# Investigation of *Toxoplasma gondii* Infection in Aborted Fetuses of Sheep Using PCR: A Study in North Khorasan Province, Iran

**DOI:** 10.1155/2020/7913912

**Published:** 2020-06-20

**Authors:** Mitra Salehi, Hosein Nezami, Hamid Reza Niazkar

**Affiliations:** ^1^Department of Medical Parasitology, Faculty of Medicine, Gonabad University of Medical Sciences, Gonabad, Iran; ^2^Department of Basic Sciences, Faculty of Medicine, Gonabad University of Medical Sciences, Gonabad, Iran; ^3^Student Research Committee, Faculty of Medicine, Gonabad University of Medical Sciences, Gonabad, Iran

## Abstract

*Toxoplasma gondii* is a zoonotic obligate intracellular protozoan parasite that infects warm-blooded animals as well as humans worldwide. The purpose of this study was to delineate the prevalence of *Toxoplasma* infection in aborted fetuses of sheep in North Khorasan province, Iran. Three hundred and ninety-nine samples of the liver (133 samples), placenta (133 samples), and brain (133 samples) from 133 aborted fetuses of sheep were collected from 2015 to 2017. The ages of aborted fetuses were higher than 120 days' gestational age in this study. According to the samples, sixteen out of 133 aborted fetuses of sheep were infected with *T. gondii*. *Toxoplasma* DNA was found in the placenta (68.75%) and liver (31.25%) samples of infected fetuses using the PCR method. The highest and lowest rates of *Toxoplasma* infection were observed during 2016 and 2017, respectively. Shirvan and Faruj provinces were recognized as the two most infected districts among others. There was a significant difference between the year and abortion rate in sheep due to infection by the *Toxoplasma* parasite (*P* < 0.05). Furthermore, no significant difference between the prevalence of *T. gondii* infection and aborted fetuses was seen (*P* > 0.05) in different areas. According to the present study, *T. gondii* infection can be one of the causes of fetus abortion of sheep in North Khorasan province, Iran.

## 1. Introduction

*Toxoplasma gondii* is a zoonotic obligate intracellular protozoan parasite from Apicomplexa phylum, which can cause toxoplasmosis in every warm-blooded vertebrate including mammals, birds, and rodents worldwide [1]. Toxoplasmosis not only can cause fetus absorption, abortion, weak, or malformed birth in sheep and goats but also is one of the significant reasons for ewe's abortion that consequently leads to economic loss [2]. These economic losses are due to the birth reduction of lamb, reduction of milk yield, and postabortion complications such as vaginal infection, fertilization delay, and infertility. The signs of toxoplasmosis in sheep fetuses are not exclusive and include general edema and fluid accumulation in cavities, which may be the result of intrauterus deaths [3]. The ordinary diagnosis of toxoplasmosis is based on histopathological examination and laboratory tests such as serologic tests and polymerase chain reaction (PCR) [4].

Additionally, toxoplasmosis is particularly important in nonimmunized women when they acquire the infection for the first time during their pregnancy since *T. gondii* is able to pass through the placenta to the fetus and causes severe complications in the fetus. Toxoplasma infection in human occurs commonly through consumption of undercooked or raw meat. Infected lamb meat is known to be of the main sources of *Toxoplasma* infection in human [5].

Studies have shown that the prevalence of *Toxoplasma* infection in aborted sheep fetuses varied between 5 and 24% worldwide and between 5 and 16% in Iran [6–14]. North Khorasan province has mild highland weather, which qualifies the region for agriculture and animal husbandry. Sheep are one of the important livestock species in this region as well as the primary source of meat, wool, and dairy products. In the current study, the prevalence of *Toxoplasma* infection in the aborted fetuses of North Khorasan province was investigated using molecular methods based on DNA detection.

## 2. Materials and Methods

### 2.1. Experimental Design

A cross-sectional retrospective survey had been conducted from 2015 to 2017, and every aborted fetus of sheep in different cities of North Khorasan, Iran, were collected (*N* = 133, no twin pregnancy). The number of samples from each city during these years are shown in Tables 1 and 2. These samples were dissected from the liver (*N* = 133), brain (*N* = 133), and placenta (*N* = 133) in all the aborted fetuses. The age of the aborted fetuses was determined by crown-rump length. Aborted fetuses were from different flocks all over North Khorasan province. It should be noted that in terms of management measures for the prevention of toxoplasmosis, these flocks are quite similar.

### 2.2. Annual Rainfall and Temperature

**Figure 1:**
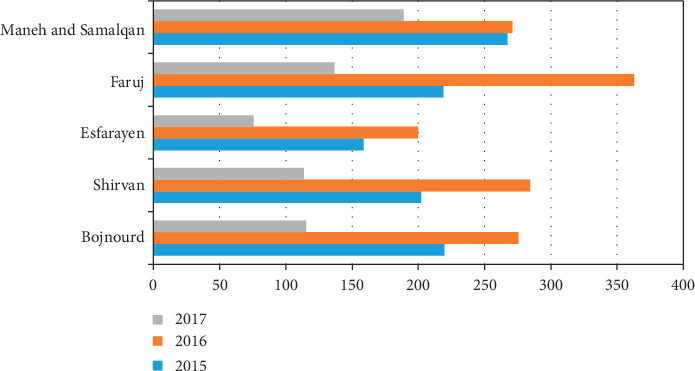
Average annual rainfall (mm) in North Khorasan cities, Iran, from 2015 to 2017.

**Figure 2:**
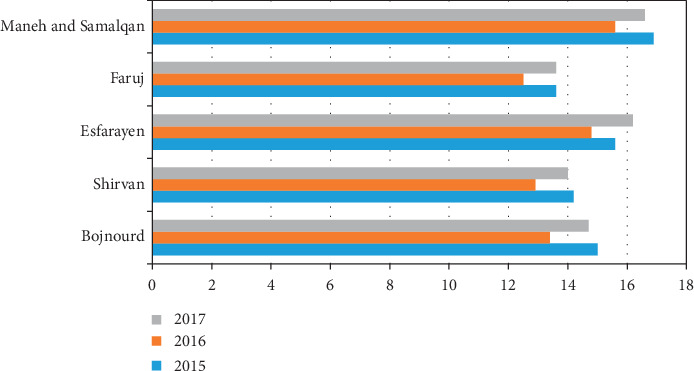
Average annual temperature (°C) in North Khorasan cities, Iran, from 2015 to 2017.

Figures 1 and 2 show the average annual temperature and rainfall in North Khorasan cities from 2015 to 2017. These data were gathered from the North Khorasan Department of Meteorology.

### 2.3. DNA Extraction

The ovine fetuses were necropsied, and the samples were collected and stored at −20°C. The phenol-chloroform and ethanol precipitation method was used for DNA extraction procedures. DNA extraction from tissues was performed with the Cinnagen DNA extraction kit (CinnaGen Company, Iran). DNA was extracted from each sample three times, and then, the concentration and purity were determined using a NanoDrop Lite Spectrophotometer (Thermo Scientific, Waltham, MA) in 260 and 280 nanometer wavelengths. Furthermore, the concentrations of the DNA samples were adjusted to 100 ng/*μ*l by diluting with double distilled water.

### 2.4. PCR Amplification Assay

In order to identify the *Toxoplasma* parasite, a polymerase chain reaction was performed. B1 gene was considered for PCR because of being highly conserved among *Toxoplasma* strains with a 35-fold repeat gene and 2,214 nucleotides in each repeat. These copies were targeted with specific primers (Tg1 (5′AAAAATGTGGGAATGAAAGAG3′) and Tg2 (5′ACGAATCAACGGAACTGTAAT 3′)) and amplified a 469-bp DNA fragment of the B1 gene. The positive control (a positive tissue sample) was kindly provided by the Mashhad University of Medical Sciences, Iran. The PCR reaction was performed in 30 *μ*l Ampliqon (Taq DNA polymerase Master mix RED, Denmark). Twenty-five microliters of Master mix were used with template DNA, 0.1 *μ*M of each primer, and distilled water. Cycles of PCR were set up as following: predenaturation step at 94°C for 3 minutes and 33 cycles (denaturation at 95°C for 35 seconds, annealing at 56°C for 45 seconds, and extension at 72°C for 1 minute) with a final elongation step for 5 minutes at 72°C. The PCR product was electrophoresed on 2% agarose gel, stained with GelRed (Biotium Inc., Hayward, CA), and visualized under the UV light.

### 2.5. Statistical Analysis

Data were analyzed using SPSS software (SPSS Inc., Chicago, IL). The chi-square test was used for the statistical analysis of qualitative data at a 5% threshold.

## 3. Results

All the aborted fetuses were older than 120 days' gestational age. *Toxoplasma* DNA was detected in 16 (12.0%) of aborted fetuses. Among these samples, *Toxoplasma* was only detected in the placenta in eleven samples (68.75%) and in both the liver and placenta in five samples (31.25%). The PCR analysis of samples using Tg1 and Tg2 was performed, and a specific 469 bp band was detected on agarose gel for positive isolates. During these three years, Shirvan city had the highest rate of aborted fetuses due to toxoplasmosis. The highest and lowest rates of *Toxoplasma* infection were observed in 2016 (23.3%) and 2017 (2.1%), respectively.

The rate of *Toxoplasma* infection was 13.7% in Shirvan and Faruj, 13.6% in Esfarayen, and 7.9% in Bojnourd and Maneh and Samalqan, respectively (*P*=0.651).

## 4. Discussion

Similar studies have been conducted in different countries worldwide; one of them showed that 9.9% of aborted fetuses of sheep in the United Arabic Emirate was due to *T. gondii* [15]. In Germany and Italy, the prevalence of *T. gondii* infection in aborted fetuses of sheep was 10 to 13%, respectively [6, 12]. In Brazil, the *T. gondii* infection was reported in 14% of aborted fetuses of sheep [8], and in Spain, the prevalence of *T. gondii* infection in aborted fetuses of sheep and goats was estimated to be 5.4 and 3.8%, respectively [10]. Finally, the prevalence of *T. gondii* infection in aborted fetuses of North America was estimated to be 20% [16].

Various investigations have been conducted across Iran, while they have shown that the prevalence of *T. gondii* infection in aborted fetuses varied between 5 and 35% depending on the province of the survey [17–20]. The prevalence of sheep fetus's abortion due to *T. gondii* infection in Khorasan Razavi province was between 5 and 16% [20, 21].

Among our 16 *T. gondii*-infected sheep fetuses, *T. gondii* was found only in placenta samples of 68.75% of fetuses, and 31.25% of fetuses had the parasite in both the liver and placenta. No *T. gondii* was detected in the brain samples of sheep fetuses infected with *T. gondii*. This may be due to the complication of DNA extraction in damaged tissue samples, such as aborted fetuses.

The prevalence of *T. gondii* in Iran depends on the climate of the area. The highest to the lowest prevalence of *T. gondii* infection was observed in temperate areas (north of Iran), droughty submontane, the cold mountainous climate of the northwest, warm and dry climate of the center, and warm and humid climate of the south of Iran, respectively [22]. The prevalence of *T. gondii* infection was estimated to be between 13.5 and 69% in sheep of Khorasan, Qazvin, and East Azerbaijan provinces, Iran [11, 20, 23]. The humid climate of East Azerbaijan and Qazvin provided a suitable environment for *T. gondii* to survive, whereas the low humidity and warm climate of Khorasan province makes the environment inappropriate for oocysts to survive [11, 20, 23]. Another study showed that the prevalence of *Toxoplasma* infection in the north of Iran is higher than other areas due to high humidity (more than 90 percent) and an average temperature of 15°C to 25°C, which provides the appropriate conditions for oocysts excreted [24]. Management conditions and factors such as presence of rodents and control of domestic cats in each farm may explain the different prevalence of *T. gondii* infection among aborted fetuses of sheep, and it is necessary to point out that mentioned surveys are not always comparable due to various diagnostic techniques and management measures of the sheep flocks [25].

The present study revealed a statistically significant relationship between the year and abortion of fetuses due to *T. gondii* infection in sheep (*P*=0.009). It should be noted that in our country, sheep are kept most of the time outdoors during the year. Therefore, the climate can affect flocks' conditions.

The highest and the lowest abortion rates in sheep fetuses due to *T. gondii* occurred in 2016 and 2017, respectively. One of the reasons for the high rate of fetus abortion of sheep due to *T. gondii* infection in 2016 may be the desirable climate for the sporulation of oocysts. Previous studies have shown that the rate of fetuses' abortion due to *T. gondii* infection increases during rainy years [26]. In Mazandaran and Gilan provinces with the average annual rainfall of 390–700 millimeters, the prevalence of *Toxoplasma* infection was estimated between 20 and 90%. Also, in Kerman province with the average rainfall of 139 millimeters, the prevalence of *T. gondii* was reported to be 24.7% [26]. As shown in Figure 1, in North Khorasan province, the annual rate of rainfall during 2016 was higher than 2015 and 2017. Additionally, the rate of *Toxoplasma* infection was also higher during 2016 with the same proportion due to the better environment for oocysts [26].

Age and immune status of fetuses affect the clinical manifestation of toxoplasmosis in pregnant ewes. During the first trimester of pregnancy, when the fetal immune system is quite immature, the probability of fetal death due to infection is higher than the rest of the pregnancy period. Midgestation infections usually result in the birth of a stillborn or weak lamb, while infection in later gestation may result in the birth of a live, clinically healthy, but infected lamb [13]. According to another study, massive abortion occurs at 110–130 days of gestation [27].

The frequency of *T. gondii* infection in the age group ≥120 days was higher than other age groups of aborted fetuses. Moreover, in our study, the age group of aborted fetuses was also higher than 120 days, which is in agreement with that of previous studies [13].

The limitation of our study was the small sample size; the authors recommend performing a larger study in various provinces of the country for more consecutive years. Also, we did not perform sequencing for the PCR products due to the limited budgets, and we suggest it for future studies.

## 5. Conclusions

This study shows that *T. gondii* infection is one of the causes of sheep fetuses' abortion in North Khorasan province, Iran. As sheep are one of the essential sources of meat and dairy products, following health-related advice, including roasting meat, is highly recommended.

## Figures and Tables

**Table 1:** Investigation of *Toxoplasma gondii* infection in aborted sheep fetuses using the molecular method from 2015 to 2017 in North Khorasan province.

Years	Results		Sum
	Positive	Negative	
2015	5 (11.6%)	38	43
2016	8 (18.6%)	35	43
2017	1 (2.1%)	46	47
Sum	14 (10.5%)	119	133

**Table 2:** Investigation of *Toxoplasma gondii* infection in aborted sheep fetuses of North Khorasan cities using PCR.

City	Results		Sum
	Positive	Negative	
Faruj and Shirvan	10 (13.7%)	63	73
Esfarayen	2 (9.1%)	20	22
Bojnourd and Maneh and Samalqan	2 (5.3%)	36	38
Sum	14 (10.5)	119 (89.5%)	133

## Data Availability

The data used to support the findings of this study are available from the corresponding author upon request.

## Additional Points

*T. gondii* infection may be one of the leading causes of ewes' abortion in North Khorasan province, Iran. There is a statistically significant relationship between the year and aborted fetuses due to *T. gondii* infection in North Khorasan. The prevalence of ewes' abortion due to *T. gondii* infection in Khorasan Razavi province was estimated to be between 5 and 16%. The prevalence of *T. gondii* in Iran varies depending on the climate of the area.
